# Terra and Aqua satellites track tiger mosquito invasion: modelling the potential distribution of *Aedes albopictus *in north-eastern Italy

**DOI:** 10.1186/1476-072X-10-49

**Published:** 2011-08-03

**Authors:** Markus Neteler, David Roiz, Duccio Rocchini, Cristina Castellani, Annapaola Rizzoli

**Affiliations:** 1 Biodiversity and Molecular Ecology Department, IASMA Research and Innovation Centre, Fondazione Edmund Mach, S. Michele all'Adige (TN), Italy; 2 Wetland Ecology Department, Doñana Biological Station (CSIC), Avda. Americo Vespucio, s/n, Seville, Spain

**Keywords:** Tiger mosquito, *Aedes albopictus*, Italy, predictive map, spatial distribution, spatial model, satellite, MODIS Land Surface Temperature, spatial entomological risk assessment

## Abstract

**Background:**

The continuing spread of the Asian tiger mosquito *Aedes albopictus *in Europe is of increasing public health concern due to the potential risk of new outbreaks of exotic vector-borne diseases that this species can transmit as competent vector. We predicted the most favorable areas for a short term invasion of *Ae. albopictus *in north-eastern Italy using reconstructed daily satellite data time series (MODIS Land Surface Temperature maps, LST). We reconstructed more than 11,000 daily MODIS LST maps for the period 2001-09 (i.e. performed spatial and temporal gap-filling) in an Open Source GIS framework. We aggregated these LST maps over time and identified the potential distribution areas of *Ae. albopictus *by adapting published temperature threshold values using three variables as predictors (0°C for mean January temperatures, 11°C for annual mean temperatures and 1350 growing degree days filtered for areas with autumnal mean temperatures > 11°C). The resulting maps were integrated into the final potential distribution map and this was compared with the known current distribution of *Ae. albopictus *in north-eastern Italy.

**Results:**

LST maps show the microclimatic characteristics peculiar to complex terrains, which would not be visible in maps commonly derived from interpolated meteorological station data. The patterns of the three indicator variables partially differ from each other, while winter temperature is the determining limiting factor for the distribution of *Ae. albopictus*. All three variables show a similar spatial pattern with some local differences, in particular in the northern part of the study area (upper Adige valley).

**Conclusions:**

Reconstructed daily land surface temperature data from satellites can be used to predict areas of short term invasion of the tiger mosquito with sufficient accuracy (200 m pixel resolution size). Furthermore, they may be applied to other species of arthropod of medical interest for which temperature is a relevant limiting factor. The results indicate that, during the next few years, the tiger mosquito will probably spread toward northern latitudes and higher altitudes in north-eastern Italy, which will considerably expand the range of the current distribution of this species.

## Background

The global spread of the invasive species *Aedes albopictus *(Skuse, 1894) (Diptera: Culicidae), the Asian tiger mosquito, is of growing public health concern in Europe. Originating from south-east Asia, it has colonized the Indo-Pacific area, Africa, the Americas and Europe over the last 30 years [1]. Global dispersal occurred through international transportation in used tires or Lucky Bamboo plants (*Dracaena sp*.) of *Ae. albopictus *eggs, which can survive drought periods for several months [2-4]. *Ae. albopictus *is proved to be a competent vector for a number of pathogens and parasites [5]. For example, it can transmit the chikungunya virus as primary vector, and the dengue virus as a vector second only to *Aedes aegypti *[6-8]. *Ae. albopictus *can also transmit West Nile virus [9-11], eastern equine encephalitis, yellow fever, La Crosse, Japanese encephalitis, Potosi, Jamestone Canyon, Tensaw, Keystone, *Dirofilaria immitis *and *D. repens *[5,8]. In Europe, there is an urgent need for intensive monitoring and development of an early warning system to predict and monitor the spread of this species, especially after the chikungunya outbreak in the Emilia-Romagna region of Italy in 2007, when more than 200 human cases were reported [10,12]. *Ae. albopictus *was introduced into northern Italy in 1990 through used tires imported from the United States [13], which led to populations being established in almost all regions of Italy [7]. In September 2010, two cases of autochthonous dengue fever were diagnosed in metropolitan France for the first time. The cases occurred in Nice, south-eastern France, where *Ae. albopictus *is established, and are evidence of dengue virus endemic transmission in this area [14].

Colonization of a new site by the tiger mosquito depends on several environmental variables [15]. Relevant parameters are winter and summer temperatures, precipitation patterns and photoperiod, all of which may influence regional differences in the dynamics and spread of this species [16,17]. The most important limiting factors in the distribution of this vector are certain air temperature thresholds as observed in a number of surveys [6,15]. In northern hemisphere winters, for example, January air temperatures have been found to limit the survival of diapausing eggs [2]. If below 0°C, egg mortality increases significantly [6,18]. In Japan, established populations can even survive mean January temperatures between 0°C and -2°C [19]; in South Korea -3°C [20]. Annual mean air temperatures have been observed to affect the seasonal dynamics and oviposition and hatching of diapausing eggs in mountain areas [21]. The threshold for population establishment is an annual mean temperature of 11°C [18,19].

Another temperature-based indicator is growing degree days (GDD), used mostly in phenology, which refers to the accumulation throughout the year of daily mean temperature values over a baseline temperature and below a cut-off temperature [22]. GDD is based on the idea that the development of, for example, an insect will only occur when the temperature exceeds a specific base temperature (T_base_) for a certain number of days. This base temperature is species specific (for *Aedes albopictus*, 11°C; [2]). GDD estimates the heat accumulation with a simple formula by taking the daily mean temperature (often simply derived from the maximum and minimum temperatures) minus the base temperature (GDD = (T_max _+ T_min_)/2 - T_base_). Before calculating the average, any temperature below T_base _is set to T_base_. Also a maximum temperature (cut-off temperature) is commonly used: values exceeding a certain temperature (usually 30°C) are set to the cut-off value as typically no further increase of the insects growth rate is expected above that temperature. GDDs are calculated year-wise from the winter minimum, e.g. nominally the 1st January of a year to the end of the year. GDD have been applied to *Ae. albopictus *with a baseline temperature of 11°C [19,22].

Several studies have been carried out using Geographic Information Systems (GIS) to generate risk maps of vector-borne diseases [23,24], including mosquito-borne diseases [25-29]. Potential establishment of *Ae. albopictus *in the United Kingdom and the Netherlands was based on mean January temperatures and annual precipitation [18,30,31].

A central problem in spatial epidemiological risk modelling is the unavailability of high resolution temperature maps for remote areas, especially in complex terrains where meteorological stations and ground surveys are usually sparsely and/or irregularly distributed and are often located only in agricultural areas. An alternative data source is remote sensing, which involves specialized satellite sensors delivering the Land Surface Temperature (LST) from space. Remote sensing technologies have been applied to *Ae. albopictus *distribution in a few studies [32-34]. New opportunities are offered by the satellites Terra (launched in December 1999, data available from 3/2000 onwards) and Aqua (launched in May 2002, data available from 8/2002 onwards). Together, they provide four LST coverages per day at various spatial resolutions, while data sets which have been aggregated over 8 and 16 days to minimize cloud effects are also available. These new satellite systems significantly improve the availability of data for scientific purposes and predictive epidemiological studies [35]. The key instrument on board both the Terra and Aqua satellites is the Moderate Resolution Imaging Spectroradiometer (MODIS). Twice a day each MODIS sensor delivers global coverages at 250 m, 500 m and 1000 m resolution in different spectral bands in a hierarchical spatial scheme [36]. The processed data are usually published less than a week after acquisition on a NASA FTP site allowing for a continuous update of the map database [37].

The aim of the present study was to identify those geographic regions in north-eastern Italy with high climatic suitability for an invasion of *Ae. albopictus *from aggregated satellite temperature data (MODIS Land Surface Temperature sensor). Besides potential distribution mapping, another goal was to verify and adapt as necessary the known air temperature thresholds for mean January temperatures, annual mean temperatures and growing degree days to thresholds derived from remotely sensed and reconstructed MODIS Land Surface Temperature data. While LST data are not directly comparable to air temperatures, which are commonly measured at 2 m above ground, it has been shown that general temperature profiles/patterns are closely correlated [38,39]. In the study of Colombi et al. [38], the obtained root mean square error (RMSE) was approximately 2°C using filtered but not further postprocessed daily MODIS LST data. Clearly, instantaneous LST measurements will deviate from a related air temperature measurement but the lower resolution of 1000 m pixel size of MODIS LST might average out effects of heterogeneous landcover on temperature. The power of a proper reconstruction of filtered daily MODIS LST data was estimated by comparing the resulting maps with entomological field data records for validation.

## Methods

### Study region

The study region is situated in the eastern-central Alps in northern Italy and includes the provinces of Trento, Bolzano and Belluno, as well as parts of the regions of Friuli-Venezia Giulia, Lombardy and Veneto (area: 24,240 km^2^; boundaries: northern latitude 46:45:00N, southern latitude 45:36:50N, western longitude 10:06:33E, and eastern longitude 12:46:30E). The study area includes the Dolomites as well as the foothills of the Alps and also small parts of the Po river plain. The northern mountainous part is characterized by several valleys, in particular the Adige river valley which has a north-south orientation and follows the south-eastern drainage pattern in this area. The study region is predominantly mountainous with a complex terrain (more than 70% of it lies 1,000 m above sea level and about 55% of the territory is covered by coniferous and deciduous forests; average mean elevation 1500 m a.s.l.; average slope 26°), and covers a large part of the Dolomites and the Southern Alps. The climate is temperate-oceanic with four main areas: sub-Mediterranean (close to Lake Garda with mild winters), subcontinental (the main valleys with more severe winters), continental (the alpine valleys) and alpine (the areas above the tree line). The annual rainfall is above 1,000 mm except for some areas in the province of Bolzano. The human population density in the region is lower than the rest of Italy. Total population is around 1,231,000 (498,000 in Bolzano Province, 519,000 in Trento Province and 214,000 in Belluno Province, as of 2008). Approximately 50% of the population is concentrated in the valley floors.

### Remote sensing and GIS analysis

We used daily MODIS LST maps [40] to derive a set of temperature-based indicators by aggregation [41-43]. The LST maps were reprojected from the original Sinusoidal projection to the Universal Transverse Mercator (UTM zone 32N) cartographic system using the MODIS Reprojection Tool (MRT 4.0; U.S. Geological Survey, 2008). In this step, the original pixel size of 1000 m was increased to 200 m in preparation for a subsequent data enhancement at the higher resolution (using nearest neighbor resampling; the cubic convolution resampling in MRT is numerically unstable at cloud fringes); furthermore, pixel values were converted from Kelvin to degree Celsius. Altogether, we processed more than 11,000 daily MODIS LST scenes from 3/2000 to 2/2009 covering the study area in a GIS framework (GRASS GIS 6.4, [44-46]). Since the pixels of the original LST maps can be cloud-contaminated, parts of the maps result void. Such incomplete maps would lead to skewed results if not gap-filled before aggregating the LST data to ecological indicators. To overcome this problem, incomplete daily LST maps were reconstructed by filling in no-data areas resulting from map filtering (bitpattern analysis of MODIS LST Quality Assessment layer) using a model-based approach. To do this, a new algorithm based on temperature gradients was recently developed [42,43], since simple interpolation without additional GIS data is not sufficient in mountainous areas, especially when larger LST map areas are devoid of information. This map reconstruction includes histogram-based outlier elimination for low temperatures in order to remove undetected clouds, as well as extraction and physical validation of the temperature gradient for each map. Despite the previously applied histogram based outlier filter, several additional filters had to be applied to the data. For the gap-filling, in case of a partially incomplete map with the number of existing pixels above the acceptable threshold, the temperature gradient is calculated based on that LST map. From this, a synthetic LST map is generated from the gradient formula. Subsequently, the lacking areas are filled in the original LST map from this synthetic map; an overall random sampling is performed on the resulting combined map, and eventually a spatial interpolation (see below for details) is performed on these points to obtain the final reconstructed LST map. In the case that the number of existing pixels is below the acceptable threshold, the same as for a completely missing map applies: these LST maps we simulated on the basis of statistical analysis of the average gradients of all available years for the given time period in order to obtain a complete time series. Let *x, y, z *be the geometrical position of a data value *w*. In general, in a volumetric 3D space the observations (*x*_*1 *_*, y*_*1 *_*, z*_*1 *_*, w*_*1*_), ... (*x*_*n *_*, y*_*n *_*, z*_*n *_*, w*_*n*_) are available where 0...*n *observations of *w *are void. The goal is to obtain *w = w*(*x, y, z*) on a regular grid. In the used approach voxels are used, i.e., raster volume pixels of (*x, y, z*). Since the desired result is a map in 2D space, the resulting *w*(*x, y, z*) values are transformed using the (*x, y, z*) points of the digital elevation model (DEM) and written out as 2D map to (*x, y, w*). The estimation of *w*(*x, y, z*) requires the fitting of a model "temperature change versus elevation change" which is based on the available LST observations and a related temperature gradient. The algorithm is using the concept of temperature gradient (i.e, the decrease of temperature with altitude) as a foundation for the reconstruction [42]. The adiabatic lapse rate which determines this gradient was calculated with linear regression applied to the individual LST/elevation maps, i.e. all LST maps with a statistically significant number of available pixels. Since air temperature is not involved, this lapse rate can be considered as a special "MODIS LST lapse rate" which is a mixture of soil (surface) temperature and near-surface temperature. In order to keep the notion of "lapse rates" for MODIS LST data, a physical connection was imposed to keep the temperature gradient slopes within reasonable limits. On the basis of the air lapse rates, the following limits were defined for MODIS LST data: "humid" land surface gradient with -0.02°C/100 m, and "dry-adiabatic" land surface gradient with -0.95°C/100 m. The former threshold value avoids to accept thermal inversion in the MODIS LST gradients which we considered hard to identify from LST data but is yet tolerant enough to permit very shallow gradients as found in some LST maps (close to "isothermal" conditions).

Hence, to obtain the reconstructed LST maps, we used altitude (elevation model in the GIS at 200 m resolution) as the explanatory variable in a volumetric splines interpolation step [42]. For all maps with a statistically significant temperature gradient the actual map gradient was selected; otherwise, a 16-day period average gradient, derived from the available nine years of daily MODIS LST data, was taken. In case of entirely missing LST maps, we simulated them on the basis of statistical analysis of the average gradients of all available years for the given time period in order to obtain a complete time series. Since the procedure of LST map reconstruction was time consuming, the maps were processed in parallel on a 128-nodes cluster.

**Figure 1:**
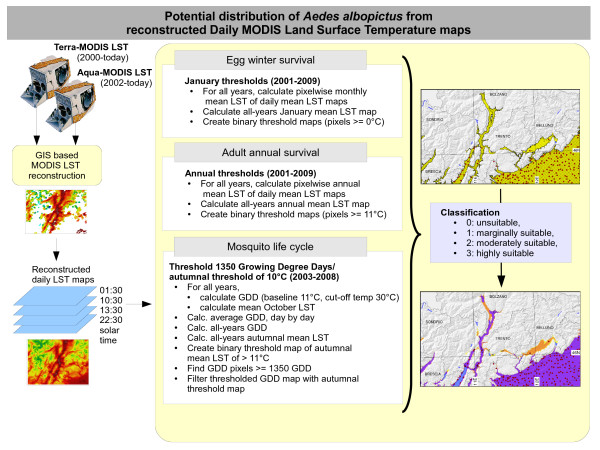
**Workflow of aggregating MODIS LST into ecological indicators for the potential distribution of *Ae. albopictus***. The original daily MODIS LST data are reconstructed mapwise and then aggregated into three different ecological indicators used as proxies to predict the potential distribution of *Ae. albopictus*.

We developed a subsequent workflow to turn the reconstructed MODIS LST data into ecological indicators which were used to assess the potential distribution of *Ae. albopictus *(see Figure 1). The following maps were derived from the reconstructed LST map set: January mean temperatures (JanT^mean^), annual mean temperatures (AnnT^mean^) and accumulated daily growing degree days corrected for autumnal mean temperature (GddT^autumn^). Each map derived from each indicator determines, respectively, winter survival of the eggs, annual adult survival and the chances to complete the mosquito life cycle [2,6,9,15,18,19].

To create the *Ae. albopictus *egg winter survival maps, we calculated all daily JanT^mean ^maps (period 2001-2009), generated the January mean temperature maps for each year from them, then calculated from them an overall JanT^mean ^average map (2001-2009). From this integrated average map we subsequently selected all pixels above the 0°C threshold [6,18] in order to obtain a binary map (threshold reached/not reached).

To obtain adult survival maps, we calculated AnnT^mean ^maps based on daily LST maps and integrated them into an all-years AnnT^mean ^average map (2001-2009). Here, areas above the 11°C threshold [18,19] were extracted in order to obtain a binary map (threshold reached/not reached).

To assess the chances of *Ae. albopictus *completing its life cycle, we calculated growing degree days [22] from daily LST maps. For this, a baseline temperature of 11°C and a cut-off temperature of 30°C were selected [2]. An average GDD series was calculated from the daily GDD maps by averaging the maps on a day-by-day basis. The resulting average GDD map series was then thresholded for 1350 GDD to identify the areas where this value was reached during the year (value selected after [19]). Since this could even occur in the winter period, we filtered the resulting map for an autumnal mean temperature threshold as a limiting factor of adult survival [47]. A threshold of 9°C average minimum air temperature indicates the end of adult activity in Trentino, but it still depends on the possibility of mosquitoes extending their reproductive season by seeking microclimates inside or near human settlements [21]. Since the average minimum temperature from LST can be lower than the air temperature in the same area [43], we decided to use the monthly average of mean daily LST temperatures at 11°C, which corresponds to the classic threshold [2]. More precisely, LST pixels with autumnal mean temperatures of > 11°C were selected as "true", otherwise "false". For the study area, we used the monthly mean LST map for October as autumnal mean temperature value, since the 11°C threshold is typically reached in this month. All pixels of the 1350 GDD map which had an autumnal mean temperature lower than this value in the respective pixel position (GddT^autumn^) were assigned a a missing data (not available) value.

The three variables obtained (JanT^mean^, AnnT^mean^, and GddT^autumn^) were integrated through map overlay by pixelwise summing up the areas of potential *Ae. albopictus *survival (i.e., egg and adult survival as well as mosquito life cycle). This resulted in four classes: pixelwise sum of 0: area not suitable; pixel-wise sum of 1: marginally suitable; pixel-wise sum of 2: moderately suitable; and pixel-wise sum of 3: highly suitable. In order to test for the potential of such climatic variables versus mere elevation for predicting *Ae. albopictus *presence we made use of additional control points [48], testing for differences in the variability of each variable (JanT^mean^, AnnT^mean^, GddT^autumn^, elevation) conditional to the presence/absence of *Ae. albopictus *by a Wilcoxon non-parametric test. The sampling points were located at several locations at Adige Valley to first characterize the mosquito distribution, so altitude of the points were at a medium and low altitude.

We assessed the relationship between winter temperatures (JanT^mean^) and altitude with linear regression and quantile regression. This analysis was carried out using R language and environment for statistical computing V2.9 (R Development Core Team, 2009) using quantile regression (quantreg package; [49]) and GRASS GIS V6.4 [44,46]. Quantile regression estimates the conditional quantiles of the distribution of a response variable in the linear model, which provides a more complete view of possible causal relationships between variables in ecological processes [50]. Here, we used quantile regression to assess the presence of microhabitats where the temperature-elevation relationship is nonlinear and differs from the normal situation. This is the case in partially shaded areas and in places close to large water bodies and also where temperature is influenced by factors other than radiation and elevation.

Data from several reports [51-53] and personal communications (Roiz D., Martini S.) confirming the presence of *Ae. albopictus *in several hundred municipalities in the study area were integrated into the database. Official reports on the presence of *Ae. albopictus *in Bolzano province were not available. As coordinates were not given in these reports, the *Ae. albopictus *observations were geocoded with *geopy *[54] using the Google Maps API according to municipality names. For this reason, the observation points are not necessarily close to the original field records but in any case located within the municipalities.

## Results

**Figure 2:**
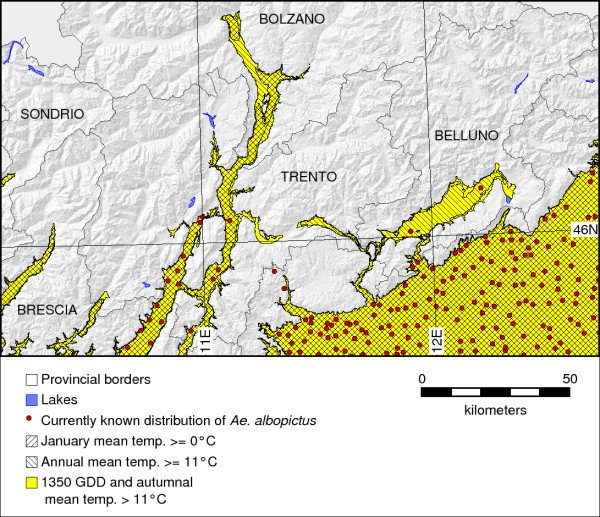
**Potential distribution areas of *Ae. albopictus *in north-eastern Italy**. The map is based on MODIS LST derived average minimum temperatures for January (JanT^mean^) integrated for the period 2001-09.

We used the three variables (JanT^mean^, AnnT^mean^, and GddT^autumn^) as predictors (or proxies) to assess the potential distribution of *Ae. albopictus *in north-eastern Italy as shown in Figure 2 (colored areas). The map shows the spatial relationship between the currently known distribution of *Ae. albopictus *(red spots; according to municipality) and the potential distribution areas by superimposing the egg winter survival area (hatched blue; JanT^mean ^> = 0°C), the adult annual survival areas (hatched red; AnnT^mean ^> = 11°C), and the areas of successful life cycle completion (yellow; growing degree days > = 1350 and autumnal mean temperature of > 11°C) (Figure 2). Vector presence as shown in this figure represents only a fraction of the true distribution of *Ae. albopictus*. The adjacent provinces of Veneto and Friuli-Venezia Giulia are densely populated with this species. The potential distribution extends the currently known distribution northwards and predicts establishment of *Ae. albopictus *up to the city of Merano and to the north of Lake Iseo (Oglio valley).

**Figure 3:**
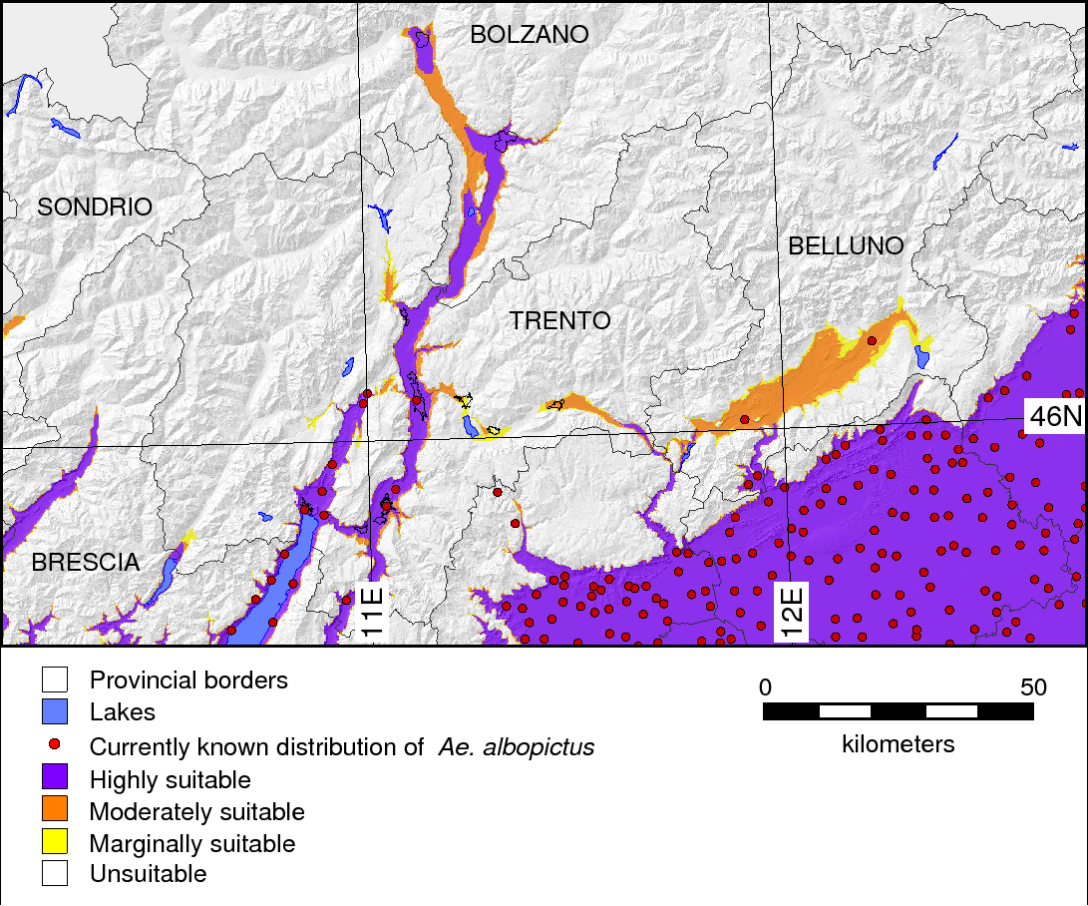
**Habitat suitability map of *Ae. albopictus *in north-eastern Italy**. The map is based on classified summary of egg winter survival, annual adult survival, and the areas of successful life cycle completion (see also explanations for Figure 2).

All three variables show a similar spatial pattern with some local differences, in particular in the northern part of the study area (upper Adige valley). Also, neither the Valsugana area nor the Belluno basin (Val Belluna) are covered by JanT^mean^, but they are supported by the other two variables (AnnT^mean ^and GddT^autumn^) (Figs. 2 and 3, see caption).

### Validation with field records

Out of all the reported municipalities where the occurrence of the species has been already proven (n = 594, provinces Belluno, Brescia, Gorizia, Padova, Pordenone, Rovigo, Trento, Treviso, Trieste, Udine, Venezia, Verona, Vicenza with 1229 municipalities in total), 590 municipalities were predicted by three variables, two municipalities were predicted by two variables and just two municipalities were not predicted at all. The contingency table (Table 1) shows the spatial overlap between the different spatialized ecological variables. The table shows that AnnT^mean ^and GddT^autumn ^coincide well (95.3%) while JanT^mean ^overlaps to a lesser extent (86.6% with GddT^autumn^, and 88.4% with AnnT^mean^). This confirms that egg winter survival is the dominant limiting factor of *Ae. albopictus *survival and establishment. Interestingly, the maximum altitude was found to be almost identical for the three variables (altitude ranges (m): JanT^mean^: min = 0.0, mean = 134.2, max = 631.0, stddev = 130.8; GddT^autumn^: min = 0.0, mean = 150.1, max = 642.0, stddev = 136.3; AnnT^mean^: min = 0.0, mean = 164.1, max = 654.0, stddev = 147.8).

**Table 1:** Contingency table showing spatial overlap between the different spatialized ecological variables (values given in percentage spatial overlap)

	JanT^mean^	AnnT^mean^	GddT^autumn^
JanT^mean^	100.0	88.4	86.6

AnnT^mean^		100.0	95.3

GddT^autumn^			100.0

### The potential distribution of *Ae. albopictus*

Figure 3 shows integration of the three variables, indicating habitat suitability for potential vector survival and establishment. The uncolored areas are those classified as unsuitable; marginally suitable areas are shown in yellow (one variable relevant), moderately suitable areas in orange (two variables relevant), and highly suitable areas in violet (three variables relevant). The map is based on classified summary of the egg winter survival area, annual adult survival area, and the areas of potential successful life cycle completion (see also explanation of Figure 2). With respect to the study area, suitability for *Ae. albopictus *in north-eastern Italy is given for the Po river plain areas and most of the Adige valley; the latter is only partially colonized as of 2010.

**Figure 4:**
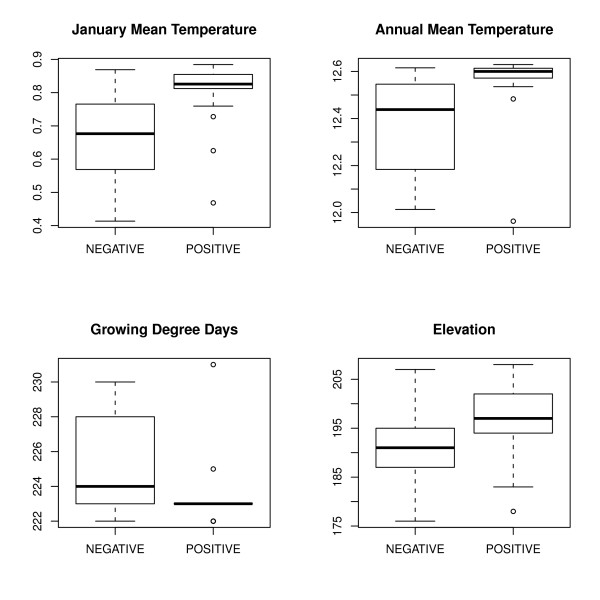
**Statistical analysis of presence or absence of *Ae. albopictus *with respect to the ancillary variables**. Differences in the values of the considered ancillary variables (JanT^mean^, AnnT^mean^, and GddT^autumn^, elevation) conditional to presence or absence of *Ae. albopictus*.

Differences in the values of the considered ancillary variables (JanT^mean^, AnnT^mean^, and GddT^autumn^, elevation) conditional to presence or absence of *Ae. albopictus *(Figure 4) were always statistically significant (Wilcoxon test, p < 0.001). Nonetheless, the range of the climate-related predictors (JanT^mean^, AnnT^mean^, and GddT^autumn^) conditional to the presence of *Ae. albopictus *was lower than that achieved considering absences (Figure 4). This means that (micro)climate conditions rather than simply elevation may shape niche characteristics promoting the optimal *Ae. albopictus *survival rate of diapausing eggs and adult mosquitoes and successful life cycle completion.

**Figure 5:**
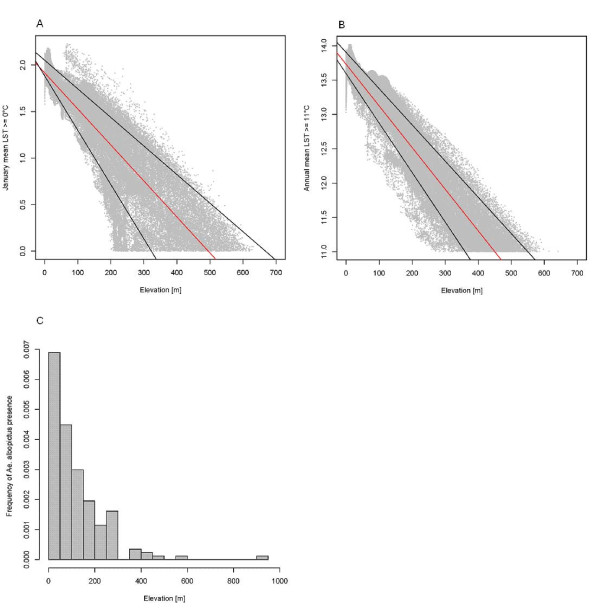
**Statistical assessment of the relationship between temperature and elevation**. Relationship between January mean LST > = 0°C and elevation (5.a), annual mean LST > = 11°C and elevation (5.b), and frequency of *Ae. albopictus *and elevation (5.c).

We analyzed the correlation between altitude and mean January LST temperatures (JanT^mean^), which determine egg winter survival, in order to assess the relationship between temperature and elevation. Figure 5a shows the linear regression line (red) and the quantile regression lines (0.10 and 0.90 quantiles, black). The linear model does not completely explain the relationship, hence data points above and below the quantile regression lines indicate micro-climates (e.g., areas with high sun exposure near Lake Garda and overly shaded areas on valley floors). The detected maximum elevation for survival based on this indicator is roughly 650 m. The histogram of known *Ae. albopictus *presence (n = 594) confirms this maximum elevation except for one outlier. The linear regression was significant (y=-0.0039x + 1.914; R^2 ^= 0.8127; p < 0.001). Using quantile regression (tau = 0.10; tau = 0.90) we found that numerous LST pixel values deviated from the linear model (pixels outside the quantile regression lines). These pixels outside the normal temperature-altitude relationship belong to areas with particular micro-climates, which may arise from terrain shadows, high exposure to sun, closeness to large bodies of water and other effects which govern the local temperature regime. For example, areas close to Lake Garda have an elevated mean winter temperature due to a latent decrease in the temperature of the lake in winter.

The same analysis was performed for annual adult survival (AnnT^mean^), and resulted in a similar pattern (Figure 5b). The linear regression was significant (y=-0.0061x + 13.731; R^2 ^= 0.9129; p < 0.001), while the same quantile regression analysis again identified a series of LST pixels outside the linear model. However, there is less heterogeneity than for JanT^mean^. A histogram analysis (Figure 5c) of the sites of known *Ae. albopictus *presence confirmed the maximum altitude of 650 m, except for an outlier municipality at around 930 m due to the political extents of the municipalities outside of the physical extents.

**Figure 6:**
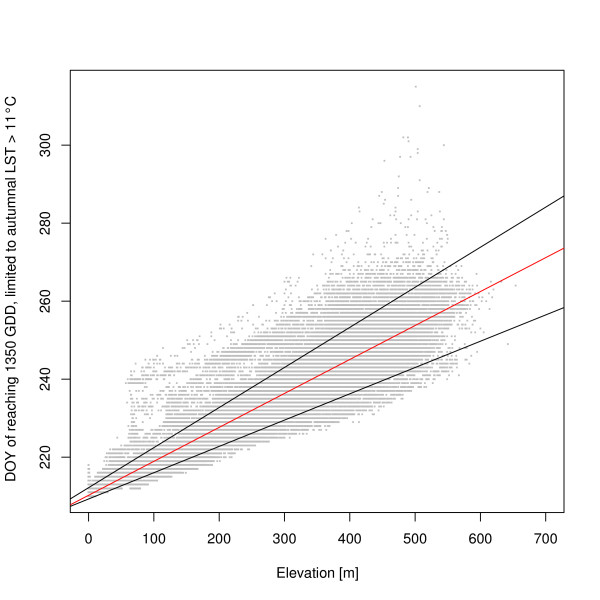
**Relationship between day of year (DOY) and elevation when 1350 GDD are reached**. Data on DOY considering all positions (pixels) with potential successful life cycle completion (1350 GDD reached with an autumnal limit of LST > 11°C) are plotted against elevation.

Figure 6 shows the relationship between the day of the year (DOY) when 1350 growing degree days are reached, with a limit of autumnal LST > 11°C, and elevation. The diagram shows the linear regression line (red) and the quantile regression lines (0.10 and 0.90 quantiles, black). The predicted maximum elevation for survival based on this indicator is roughly 650 m.

The variability in reaching the survival threshold increases with higher elevation. The predicted maximum elevation for survival based on this indicator is roughly 650 m. The linear regression was significant (y = 0.0871x + 210.23; R^2 ^= 0.8962; p < 2.2e-16), and also here quantile regression analysis identified a series of LST pixels outside the linear model.

**Figure 7:**
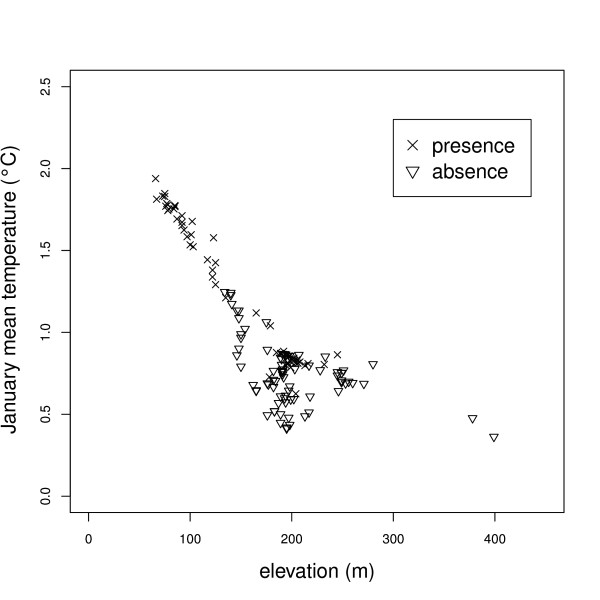
**Relationship between the altitude, JanT^mean ^and the current distribution of *Ae. albopictus***. The absence/presence data and elevations were obtained from traps. The complete data set for this figure is included with the manuscript as additional file 1.

In order to assess the probability that LST outliers dominate the result of mosquito winter survival, we analyzed trap data along with the related MODIS LST pixel data. Figure 7 shows the relationship between temperature, trap elevation and mosquito survival. In general, with increasing altitude the number of positive traps decreases. However, in the range of 170-300 m a mixed cluster of traps with *Ae. albopictus *being absent and present is observed. The mixed presence and absence clearly indicate that the microclimatic situation is differentiated through the use of MODIS LST data. In the hypothetical case of systematic LST outliers we would rather observe a regular patterns which is not found.

## Discussion

In the present study, reconstructed daily MODIS Land Surface Temperature data were used to the estimate the potential distribution areas of the tiger mosquito *Ae. albopictus *in north-eastern Italy. We developed a methodology which allows to capture certain abiotic conditions, crucial for insect survival and establishment, to be described precisely based on three ecological variables derived from daily satellite data. Rather than interpolating temperature ground measurements which is fairly difficult to interpolate to temperature maps in mountainous areas we used LST data which are intrinsically spatialized as satellite images. Prior to derive indicators from the LST data we optimized those by applying outlier detection and gap-filling. We used this method in a geographic area of northern Italy which is currently invaded by *Ae. albopictus*. The study area is characterized by a particularly complex terrain which gives rise to many micro-climate zones. Using high resolution time series from two MODIS sensors on board the Aqua and Terra satellites in a GIS framework it is now possible to obtain detailed predictive maps of potential future insect establishment and spread.

A known method of deriving environmental indices is Temporal Fourier Analysis (TFA) of satellite imagery where multi-annual data are aggregated by a Fourier transform to find temperature or vegetation patterns [55-57]. The Temporal Fourier analysis (TFA) delivers a set of sine curves (harmonics), which are of different frequencies, amplitudes and phases. The set of all curves sums up to the original time series signal. Its application to MODIS LST data has been reported to introduce errors of up to 30% in the estimation of the amplitudes and phases of the Fourier harmonics [57]. In fact, contrary to the method proposed in this paper, inter-annual variations are to some extent lost in TFA since multi-annual data are aggregated. Hence, our approach is more detailed with respect to recent publications where remotely-sensed LST data were also applied to assess the distribution of *Ae. albopictus*. For example, the European Centre for Disease Prevention and Control [32] used MODIS 8-day LST composite images at 1000 m resolution, which had been enhanced using a spatial and temporal spline transformation prior to a Fourier transformation, resulting in Fourier-processed data layers as well as aggregated minimum, maximum and diurnal change variables. A similar approach was used by Carbajal de la Fuente et al. [58].

Unlike these approaches, in our study we used daily gap-filled MODIS LST data while the aforementioned studies were based on the 8-day period MODIS LST data product. Since our data have a higher resolution of 200 m [43] they allowed us to get a more detailed picture of the situation regarding micro-climates. This new data set performed well for the three derived variables owing to its higher spatial and temporal resolution. The use of remotely sensed, gap-filled temperature data as an alternative to maps from interpolated meteorological station data offers the potential to extend the assessment from regional to even a subcontinental scale. The use of reconstructed MODIS land surface temperature data contributes significantly to a more precise picture of the new areas of potential establishment and spread of *Ae. albopictus*, which could not have been easily obtained from interpolation of temperature data from meteorological station data. This approach using MODIS LST data, will be useful for public health agencies and research institutions wishing to implement early warning surveillance systems, given that control measures are most effective at the preliminary phase of colonization of this vector. With new MODIS LST data being continuously processed, the JanT^mean ^indicator map can be obtained already in the beginning of February of the actual year in order to predict the winter survival rate of the eggs. For the indicator GddT^autumn ^there are two possibilities: during the year parametrized GDD curves can be applied to each pixel time series which is then updated weekly. The full, real GddT^autumn ^can then be generated in the beginning of November. The AnnT^mean ^indicator is available in the beginning of January of the following year.

Since we consider temperature to be the most important limiting factor in our study area, our work is based on three temperature indicators (JanT^mean^, AnnT^mean^, and GddT^autumn^) which best describe the distribution of *Ae. albopictus*. Altitude is not considered a limiting factor, as (micro)climatic conditions and niche characteristics are rather determined by temperatures that condition adult and egg mortality due to low temperatures below the thresholds [2,19]. We therefore generated and integrated these three LST based indicators and validated them at a regional scale with field data obtained from northern Italian *Ae. albopictus *populations. We consider this approach to be valid for all other areas where precipitation is not a limiting factor, i.e. where the mean annual precipitation is above 500 mm, as is the case in most European areas, except for some parts of southern Spain, southern Italy, southern Greece, Turkey and Bulgaria [9].

For all the three temperature related variables, the suitability map showed a coherent overlap with field data (see Figure 2). JanT^mean ^affects the overwintering ability of the tiger mosquito since below a mean temperature of 0°C they will suffer from considerable egg winter mortality. In some areas (such as Bolzano, the Valsugana Valley and the upper Adige valley) where the annual and autumnal conditions allow *Ae. albopictus *adults to survive, winter egg survival is not possible according to the prediction obtained in our model. In these areas, therefore, the recurring presence of tiger mosquitoes may only result from ongoing continuous reintroduction of the species, as already demonstrated in Japan [19]. Hence, the risk of establishment is reduced in these areas. In fact, our approach is able to discriminate between areas at risk of seasonal introduction of *Ae. albopictus *(based on annual mean temperatures) or at risk of establishment and spread over several years (based on January mean temperatures that could be highly useful to understand in what areas the populations of the tiger mosquito could (or not) overwinter [19,59]).

The AnnT^mean ^values condition mosquito activity during the summer season and the relative abundance inside the potential distributional areas, its threshold of 11°C being consistent with zero development of this mosquito and the northern distribution limit in Japan [19]. The potential distribution areas derived from thresholding of AnnT^mean ^(Figure 2) which shows where this species can survive when the mean annual LST temperature is over 11°C differ slightly from those derived from GddT^autumn ^maps. Almost all of the valley floor areas and the Po river plain are resulting as suitable from the map.

The GddT^autumn ^variable (Figure 2) has the largest spatial extent and is hence the least limiting factor. In some areas (Valsugana Valley and north of the Santa Croce Lake near Belluno) there are areas with suitability indicated only by GddT^autumn^. In these areas, the existence of favorable microclimates in urban areas could allow establishment of the species, but we consider that an extensive spread of this invasive species is improbable due to an expected high mortality of diapausing eggs in winter.

The integrated map shows a potential future spread of *Ae. albopictus *towards areas with higher latitude and elevation than the current distribution (violet areas with no red spots in Figure 3) and may therefore be used as an "early warning system" to prevent the spread of this species. When comparing the three MODIS LST based indicators, the JanT^mean ^indicator is the most limiting as it conditions the survival of eggs in winter and hence the overwintering of populations (Table 1; Figure 2). The advantage of using remotely sensed temperature maps is that they intrinsically obtain microclimatic features (certainly with the limitations of the spatial resolution) which affect the distributional pattern of this species (i.e. the local contribution of valley orientation, insolation time, heterogeneous land cover, and the highly variable geomorphology due to a complex terrain). To obtain a similar detailed view from interpolated meteorologic station data is rather difficult due to the commonly non-representative distribution of the stations. Our approach proved that considering satellite based temperature maps is revealing a more detailed picture than simply using altitude as proxy in determining the distribution of this species. Microclimatic areas have to be taken into account when studying the influence of abiotic factors on the distribution of a species. Figure 7 illustrates in the range of 200 m altitude that altitude does not allow to discriminate between *Ae. albopictus *being present or absent since a mixed pattern is found in this range. We observed that the areas where *Ae. albopictus *survives are at low altitude, corresponding to the main Alpine valley floors, while areas above 650 m do not fall into the potential distribution areas. We tried to define this elevation limit for the potential distribution of the tiger mosquito in this alpine area and similar ones, in this case around 650 m, determining it on the basis of land surface temperatures. This prediction was confirmed in 2010, when we detected *Ae. albopictus *at 647 m in Val di Tenno (Roiz et al., unpublished results).

More work aimed at collecting field observations is therefore necessary to validate our predictions, especially to verify if this population is (or not) overwintering or if the presence of this species is due to continuous reintroduction.

In future work, we aim to relate the results of this detection (and several others at this range of altitudes) to the three different LST variables.

## Conclusions

In this paper we demonstrated the power of using reconstructed daily MODIS Land Surface Temperature map time series to derive climate-based variables (January temperatures, annual mean temperatures and growing degree days filtered for an autumn threshold temperature) used as validated predictors to assess the expected potential distribution of *Ae. albopictus *in north-eastern Italy. The results show that i) LST data from satellites can be used to predict areas of potential short term invasion of the tiger mosquito with considerable accuracy and ii) during the next few years and in a scenario of increasing temperatures, the tiger mosquito might spread toward northern latitudes and higher altitudes in several valleys located in north-eastern Italy.

Future research should be aimed at extending and validating the prediction obtained with the proposed model to a larger scale, including the western Italian Alps. Furthermore, we envision that gap-filled daily MODIS LST data could be applied to risk assessment and spatial modelling of other animal species (e.g. other arthropod species) under different global change scenarios and to robustly evaluate the effect of temperature changes in affecting animal and plant current and future distribution.

## List of abbreviations

DOY: Day Of Year; FTP: File Transfer Protocol; GDD: Growing Degree Days; LST: Land Surface Temperature; MODIS: Moderate Resolution Imaging Spectroradiometer; MRT: MODIS Reprojection Tool.

## Competing interests

The authors declare that they have no competing interests.

## Authors' contributions

MN, DaR and CC originally formulated the ideas presented in this paper. MN reconstructed the MODIS sensor data for the study and was in charge of data analysis. MN, DaR, DuR, CC and AR wrote the initial draft of this manuscript and all authors contributed extensively to the preparation and revision of the final manuscript.

## Authors' information

Markus Neteler is head of the GIS and Remote Sensing unit of the Department of Biodiversity and Molecular Ecology, IASMA Research and Innovation Centre at the Fondazione Edmund Mach (FEM), Trento, Italy. His main research interests are remote sensing for environmental risk assessment, spatial modeling and Free Software GIS development.

## Supplementary Material

Additional file 1 ***Aedes albopictus *presence/absence data in North-eastern Italy for 2008/2009**. Complete data set of Figure 7 showing the relationship between altitude, JanT^mean ^and the current distribution of *Aedes albopictus *(absence/presence data from traps; altitude from trap elevations). The column meanings are: ID: Identification number; TRAPID: Trap identification code; YEAR: Year of observation; TRAP_TYPE: Trap type; MUNICIPALITY: Municipality of trap position; ADDRESS: Address of trap position; AE_PRESENCE: *Ae. albopictus *presence or absence; AE_TOTAL_EGGS: *Ae. albopictus *total number of eggs; HUMPOP2007: Human population; JAN_TMEAN: January mean temperature; ELEVATION: Trap elevation. Click here for file
